# Efficient Genome Editing in *Setaria italica* Using CRISPR/Cas9 and Base Editors

**DOI:** 10.3389/fpls.2021.815946

**Published:** 2022-01-13

**Authors:** Zhen Liang, Yuqing Wu, Lingling Ma, Yingjie Guo, Yidong Ran

**Affiliations:** ^1^School of Life Sciences, Shanxi University, Taiyuan, China; ^2^Institute of Fundamental and Frontier Sciences, University of Electronic Science and Technology of China, Chengdu, China; ^3^Shenzhen Polytechnic, Shenzhen, China; ^4^Genovo Biotechnology Co. Ltd, Tianjin, China

**Keywords:** CRISPR/Cas9, genome editing, base editing, *Setaria italic*, herbicide resistance

## Abstract

The genome editing toolbox based on CRISPR/Cas9 has brought revolutionary changes to agricultural and plant scientific research. With the development of stable genetic transformation protocols, a highly efficient genome editing system for foxtail millet (*Setaria italica*) is required. In the present study, we use the CRISPR/Cas9 single- and multi-gene knockout system to target the *SiFMBP*, *SiDof4*, *SiBADH2*, *SiGBSS1*, and *SiIPK1* genes in the foxtail millet protoplasts to screen out highly efficient targeted sgRNAs. Then, we recovered homozygous mutant plants with most of the targeted genes through an *Agrobacterium*-mediated genetic transformation of foxtail millet. The mutagenesis frequency in the T_0_ generation was as high as 100%, and it was passed stably on to the next generation. After screening these targeted edited events, we did not detect off-target mutations at potential sites. Based on this system, we have achieved base editing successfully using two base editors (CBE and ABE) to target the *SiALS* and *SiACC* genes of foxtail millet. By utilizing CBE to target the *SiALS* gene, we created a homozygous herbicide-tolerant mutant plant. The current system could enhance the analysis of functional genomics and genetic improvement of foxtail millet.

## Introduction

Foxtail millet (*Setaria italica*) is a very important food crop in Asia and Africa. Compared with other common grains, foxtail millet is rich in protein, vitamins, minerals, and fiber, and the content of some macroelements and microelements is also relatively high, which makes it a nutritious crop (Han et al., 2015). In addition to its excellent nutritional value, foxtail millet also tolerates various abiotic stresses well, has good resistance to pathogens, and exhibits high utilization of nitrogen. It is an environmentally friendly crop that uses less water or fertilizers, and it is a strategic reserve crop to deal with environmental changes such as drought (Lata et al., 2013). Foxtail millet is a diploid crop with a small genome (Zhang et al., 2012). Genome sequencing has been completed, and it has gradually been developed into a xerophytic C4 cereal model crop (Doust et al., 2009). Recently, the Wang group developed a foxtail millet variety “*xiaomi*” with small size and short life cycle by screening the traditional EMS mutant library, which has sped up the use of foxtail millet as a model plant (Yang et al., 2020). However, current basic research on foxtail millet is extremely weak, and there is a lack of effective research methods. The emergence and development of genome editing technology provides new opportunities and hope for relevant research on foxtail millet.

CRISPR/Cas9 technology is the most effective gene editing system. The system is mainly composed of two parts: single guide RNA (sgRNA) and Cas9 endonuclease (Cong et al., 2013). With the help of sgRNA, specific targets that contain the protospacer-adjacent motif (PAM) are identified and cut, which results in double-strand breaks (DSB) in the host genome, that trigger non-homologous end joining (NHEJ), microhomology-mediated end joining (MMEJ), or homology-directed repair (HDR) pathways to repair the DNA damage. The NHEJ and MMEJ pathways usually generate targeted insertions/deletions (indels), but the HDR pathway introduces precise gene replacements or insertions (Chen et al., 2019; Tan et al., 2020). Base editing was developed based on the CRISPR/Cas9 system, the Cytosine Base Editor (CBE) and Adenine Base Editor (ABE), which is composed of a nickase Cas9 (nCas9) and a cytosine or adenine deaminase. This editing can achieve precise replacement of bases at specific sites (C to T or A to G) without producing double-strand breaks and then cause the substitution of amino acids at specific sites that changes the function of genes (Komor et al., 2016; Gaudelli et al., 2017). In the past 10 years, CRISPR/Cas9 system and the derived base editing systems have been used to do functional genomic studies, trait improvement and breeding in common crops such as rice, maize, wheat, and tomato (Shimatani et al., 2017; Zong et al., 2018; Zhang et al., 2019). However, a highly efficient genome editing system in foxtail millet has not been established yet, although there are a few relevant reports (Lin et al., 2018; Cheng et al., 2021; Zhang et al., 2021). Genome editing in foxtail millet was firstly demonstrated to disrupt *SiPDS* gene in transient protoplast assay, but no stable mutant was obtained (Lin et al., 2018). Cheng et al. (2021) generated a haploid inducer line by using CRISPR/Cas9 technology to target the *SiMTL* gene, with a mutagenesis efficiency of 26%. However, there are still no reports on the application of base editing in foxtail millet.

Here, we report the development of a highly efficient genome editing system with single, multiple gene knockouts and single base substitution using the CRISPR/Cas9 system, cytosine and adenine base editing systems in foxtail millet based on an *Agrobacterium*-mediated transformation. Six genes related to agronomic traits were selected as targets to establish the CRISPR/Cas9 knockout system. Foxtail millet bran protein (FMBP) is a peroxidase that has anti-colon cancer effects (Shan S. et al., 2014). Maize DNA-binding with one finger 1 (Dof1) increases carbon and nitrogen assimilation under low-nitrogen conditions (Kurai et al., 2011). In subsequent experiments, the homologous gene *SiDOf4* was chosen (Zhang et al., 2017). The Betaine aldehyde dehydrogenase 2 (BADH2) inhibits the synthesis of 2-acetyl-1-pyrroline, which is a major component in fragrance (Chen et al., 2008). The Granule bound starch synthase 1 (GBSS1) is responsible for the synthesis of amylose, and interruption of the *GBSS1* gene resulted in waxy maize (Gao et al., 2020). Inositol-pentakisphosphate 2-kinase 1 (IPK1) catalyzes the final step in phytate biosynthesis, which is an ideal target for phytate reduction (Shukla et al., 2009). For base editing experiments, the acetolactate synthase (ALS) and acetyl-coenzyme A carboxylase (ACC) that associated with herbicide tolerant were chosen as primary target genes (Li et al., 2018; Zhang et al., 2020). Homozygous or bi-allelic mutants were obtained in the T_0_ generation, and the targeted mutations were transmitted to the next generation.

## Materials and Methods

### Single Guide RNA Design and Vector Construction

For single gene knockout, the potential sgRNAs were designed using CRISPR-P online software^1^ and the top two sgRNAs were selected based on the mismatch numbers with other potential off-target sites. Pairs of oligonucleotides of sgRNAs were synthesized, annealed, and cloned into corresponding *Bsa*I-digested pHUE411 plasmid (Xing et al., 2014). To construct the pH-CBE vector, the codon-optimized NLS-APOBEC1-XTEN-nCas9-UGI-NLS fusion protein were synthesized commercially and cloned into the pHUE411 backbone via Gibson Assembly to replace the Cas9 gene. To construct the pH-ABE, the codon-optimized ecTadA WT/7.10 deaminase and three copies of the NLS were synthesized commercially. The ecTadA WT/7.10, nCas9 and 3*NLS were then sequentially cloned into pHUE411 backbone via Gibson Assembly. For base editing experiments, the sgRNAs involved in herbicide resistance were selected and cloned into *Bsa*I-digested pH-CBE and pH-ABE, respectively.

To construct the multiplex genome editing vector using multicomponent transcriptional unit (hereafter MCTU) system to express the sgRNAs that targeted *SiDOf4*, *SiBADH2*, *SiGBSS1*, and *SiIPK1*, three fragments were prepared to assemble the sgRNAs array. MT1-Si9-BsF, MT1-Si9-F0, and MT0-BsR2 were used to amplify fragment 1 from pCBC-MT1T2 plasmid. MT2-Si12-BsF2, MT2-Si12-F0, and MT0-BsR3 were used to amplify fragment 2 from pCBC-MT2T3 plasmid. MT3-Si14-BsF3, MT3-Si14-F0, MT4-Si15-R0, and MT4-Si15-BsR were used to amplify fragment 3 from the pCBC-MT3T4 plasmid. The three fragments were then inserted into the pHUE411 vector using the Golden Gate method (Xing et al., 2014).

To construct the multiplex genome editing vector using polycistronic tRNA-gRNA (hereafter PTG) to express the sgRNAs that targeted *SiDOf4*, *SiBADH2*, *SiGBSS1*, and *SiIPK1*, five fragments were prepared to assemble the sgRNAs array. L5AD5-F/gRSi9-R, gRSi9-F/gRSi12-R, gRSi12-F/gRSi14-R, gRSi14-F/gRSi15-R, and gRSi15-F/L3AD5-R primer pairs were used to amplify five fragments from pGTR plasmid. Then the five fragments were ligated using the Golden Gate method. The products were amplified using the S5AD5-F/S5AD5-R primer pair, digested by *Fok*I and inserted into *Bsa*I-digested pGREB32 plasmid (Xie et al., 2015).

To construct the multiplex genome editing vector using Csy4 separated sgRNA arrays (hereafter Csy4) that targeted *SiDOf4*, *SiBADH2*, *SiGBSS1*, and *SiIPK1*, five fragments were prepared to assemble the sgRNAs array. oPvUbi1/CSY_gRNASi9, REP_gRNASi9/CSY_gRNASi12, REP_ gRNASi12/CSY_gRNASi14, REP_gRNASi14/CSY_gRNASi15, and REP_gRNASi15/CSY_term primer pairs were used to amplify five fragments from pDirect-25H plasmid. Then, the resulting fragments were inserted into the pDirect-25H vector using the Golden Gate method (Cermak et al., 2017). The resultant constructs were confirmed by Sanger sequencing and further used for protoplast transfection or *Agrobacterium*-mediated transformations.

### Protoplast Transfection

Foxtail millet protoplast transfection was performed as previously described (Shan Q. et al., 2014) with slight modifications. Mature seeds of foxtail millet were sterilized and grown in 1/2 MS solid medium at 26°C with a photoperiod of 16 h light (full spectrum LED light with ∼150 μmol m^–2^ s^–1^) and 8 h dark for 16–20 days. The stems separated from seedlings were cut in cross section and incubated in enzyme solution (1.5% Cellulase, 0.3% Macerozyme, 0.6 M mannitol, 10 mM MES at pH 5.7, 1 mM CaCl_2_, and 0.1% BSA) for 5 h with gentle shaking at 80 rpm. The stems of young seedlings were short, and usually about 10 seedlings were needed for each transformation. After digestion, the enzyme solution was filtered through a 45 μM nylon filter and discarded. Then, the digested tissues were washed 2–3 times with 50 mL W5 solution (154 mM NaCl, 125 mM CaCl_2_, 5 mM KCl, and 4 mM MES) and fresh protoplasts were released from the cutting edge. Protoplasts were collected by centrifuge at 70 *g* for 3 min and resuspended in MMG solution (0.4 M mannitol, 15 mM MgC_*l2*_, and 4 mM MES). Twenty μg plasmids were delivered into 200 μL protoplasts using PEG-mediated transfection and incubated for 20 min in the dark at room temperature. Transfected protoplasts were washed with W5 and collected by centrifuge at 70 *g* for 3 min. Protoplasts were incubated in W5 in the dark at RM temperature. After 48 h incubation, the protoplasts were collected and genomic DNA was extracted using the CTAB method. In order to guarantee the success and a relative high transfection efficiency, a GFP plasmid was used for transfection as a positive control (Supplementary Figure 1).

### *Agrobacterium*-Mediated Stable Transformation

The resultant constructs were transformed into wild type *Agrobacterium tumefaciens* strain EHA105 using a freeze/thaw method and selected on LB medium that contained kanamycin (50 mg/L) and rifampicin (25 mg/L). The clones were screed by colony PCR. Then, the plasmids in the positive clones were extracted and confirmed by Sanger sequencing. Transformation was carried out following Sood et al. (2020) with minor modification. Briefly, mature seeds of two varieties were de-husked, sterilized, and plated on callus induction medium at 26°C in the dark (i.e., Yugu1 and *xiaomi*; Both seeds obtained from the stock of Prof. Xingchun Wang’s lab, College of Life science, Shanxi Agricultural University, China. All plant materials were provided free of charge and used for research only). After 3 weeks, the buds were removed and the induced calli were transferred to fresh callus induction medium for three rounds of subculture (2–3 weeks for each round) to obtain embryonic calli for transformation. Embryogenic calli were incubated with *Agrobacterium* strain EHA105 for 15 min, dried on filter paper and incubated in the dark for 3 days. The transformed calli were then selected with hygromycin (40 mg/L) for 1 month. Thereafter, surviving calli were regenerated at regeneration medium with hygromycin (15 mg/L) at 26°C with a photoperiod of 16 h light and 8 h dark for 6–8 weeks. The regenerated shoots were then plated on the rooting medium with hygromycin (20 mg/L). After 2 weeks, the transgenic plantlets were available for genotyping.

### Genotyping and Detection of Mutations

Mutations that occurred in protoplasts assay were detected by two methods: PCR/T7EI assay and deep amplicon sequencing. The PCR/T7EI assay was conducted as described previously (Shan Q. et al., 2014). For deep amplicon sequencing, two rounds of PCR were performed as described previously (Liang et al., 2018). In the first round PCR, PCR products that contained the target site were amplified using gene-specific primers. In the second round PCR, primer sets with forward and reverse barcodes were used to limit the product size to within 200 bp for library construction and Illumina sequencing. NGS reads were analyzed using the Cas-Analyzer.^2^ Mutation that occurred in transgenic plants were detected by PCR and subsequent Sanger sequencing. The sequencing chromatograms were deciphered by the DSDecode web tool (Liu et al., 2015).

### Herbicide Resistance Test Assay

To detect the effective level of nicosulfuron herbicide, seedlings in the greenhouse of wild type *xiaomi* at the four-leaf stage were sprayed with 30 g ai ha^–1^ concentrations of nicosulfuron, which was an effective concentration of nicosulfuron as determined by the obvious death of wild-type seedlings. Base-edited plant T_1_-1-12 with homozygous P170A mutation in ALS alleles were then used in the herbicide-resistance assay. Seedlings in the greenhouse at the four-leaf stage were treated with nicosulfuron at a concentration of 30 g ai ha^–1^. Photos of plants were taken about 4 weeks after the herbicide treatments. At least three biological replicates were used for each treatment.

## Results

### Development of CRISPR-Mediated Efficient Gene Knockout of Foxtail Millet

**FIGURE 1:**
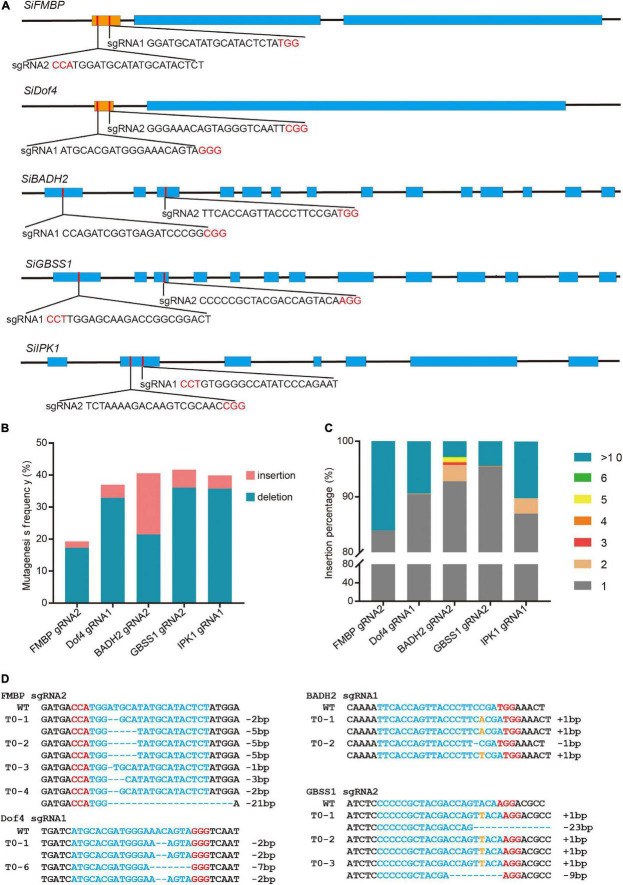
Targeted mutagenesis in foxtail millet using the CRISPR/Cas9 system. **(A)** Schematic structures of the *SiFMBP*, *SiDof4*, *SiBADH2*, *SiGBSS1*, and *SiIPK* genes. The blue and brown boxes indicated exons and uORF, respectively. Two sgRNAs were designed to target each gene. The PAM sequences were highlighted in red. **(B)** Mutagenesis frequencies of *FMBP* sgNRA2, *Dof4* sgRNA1, *BADH2* sgRNA2, *GBSS1* sgRNA2, and *IPK1* sgRNA1 in transient protoplasts assay were analyzed by next generation sequencing. **(C)** Percentages of insertion mutation with different lengths tested in five sgRNAs using transient protoplast assay. **(D)** Sanger sequence analysis of representative T_0_ mutants induced by *FMBP* sgRNA2, *Dof4* sgRNA1, *BADH2* sgRNA2, and *GBSS1* sgRNA2. The protospacer and protospacer-adjacent motif (PAM) sequences were highlighted in blue and red, respectively. Nucleotides inserted at the target sites were labeled orange.

Due to the laborious and time-consuming tissue culture procedure, the protoplast assay system was established to test the activities of the sgRNAs that we designed. We first designed two sgRNAs to target each of the five endogenous genes, *SiFMBP* (*Seita*.*5G463200*), *SiDof4* (*Seita*.*1G303300*), *SiBADH2* (*Seita*.*6G151100*), *SiGBSS1* (*Seita*.*4G022400*), and *SiIPK1* (*Seita*.*3G025600*) (Figure 1A). The CRISPR-encoding constructs were introduced into protoplasts of foxtail millet using PEG-mediated transfection system that we established following the protocol described previously (Shan Q. et al., 2014) (Supplementary Figure 1). Genomic DNA was extracted from the protoplasts incubated for 48 h after transfection and analyzed using a PCR/T7EI method (Supplementary Table 1). The mutagenesis efficiencies of the six target sites were determined by the band densities (Supplementary Figure 2). NGS was further performed to determine the specific mutation rate and pattern (Figure 1B and Supplementary Figure 3). In addition to the small indel mutations, there were a few mutation events with large fragments (>10 bp) inserted at the five sites (Figure 1C). Among them, the proportion of large insertion at the targets sites of *FMBP* sgNRA2 and *Dof4* sgRNA1 accounted for a relatively high rate (16.11 and 9.42%, respectively). Based on the sequence analysis, the insert fragments were all derived from the plasmids used for editing (Supplementary Figure 4).

Next, the sgRNAs (*FMBP* sgRNA2, *Dof4* sgRNA1, *BADH2* sgRNA2, and *GBSS1* sgRNA2) with a higher mutagenesis frequency that had been confirmed with protoplast transient assay for each target gene were selected to produce mutated plants using *Agrobacterium*-mediated transformation. We found that the mutation efficiencies of each target site in T_0_ generation can be up to 100% (Figure 1D and Table 1). For *FMBP* sgRNA2, 2 out of 7 T_0_ lines were confirmed as homozygous mutants and the remaining five lines had bi-allelic mutations. Most mutation types were small deletions within 5 bp, except for line #4 which had -21 bp deletion. This longer deletion may be due to MMEJ repair using the “TGG” micro-homology sequence. For *Dof4* sgRNA1, 6 out of 7 T_0_ lines were homozygous mutants and the remaining one had bi-allelic mutations. All six homozygous mutants exhibited the same mutation types with -2 bp deletion, which was consist with deep sequencing results from the protoplast assay that showed the -2 bp deletion at the target site was the most frequent mutation type (Supplementary Figure 3). For *BADH2* sgRNA2, 5 out of 6 T_0_ lines had desired mutations, in which two of them were homozygous mutants, and the others were bi-allelic mutants. For *GBSS1* sgRNA2, we obtained one homozygous and two bi-allelic mutants. Furthermore, the knockout construct with *Dof4* sgRNA1 was also delivered into another foxtail millet variety (cv *xiaomi*), two T_0_ lines were regenerated and both of them contained the desired target homozygous or bi-allelic mutations (Table 1).

**TABLE 1:** Summary of T_0_ plant characterization.

Target site	Varieties	Genome editing tool	No. of T_0_ plants tested	Mutated T_0_ lines: number, ratio (%)	Homozygous T_0_ lines: number, ratio (%)	Bi-allelic T_0_ lines: number, ratio (%)	Heterozygous T_0_ lines: number, ratio (%)
*FMBP* sgRNA2	Yugu1	SpCas9	7	7, 100%	2, 28.6%	5, 71.4%	N.D
*Dof4* sgRNA1	Yugu1	SpCas9	7	7, 100%	6, 85.7%	1, 14.3%	N.D
*BADH2* sgRNA2	Yugu1	SpCas9	6	5 83.3%	2, 33.3%	3, 50%	N.D
*GBSS1* sgRNA2	Yugu1	SpCas9	3	3, 100%	1, 33.3%	2, 66.7%	N.D
*Dof4* sgRNA1	*xiaomi*	SpCas9	2	2, 100%	1, 50%	1, 50%	N.D
*ALS* sgRNA	*xiaomi*	CBE	2	1, 50%	N.D	N.D	N.D
*ACC* sgRNA	*xiaomi*	ABE	13	4, 30.8%	2, 15.4%	N.D	2, 15.4%

High heritability is of great significance in breeding. Three independent mutants that targeted *FMBP* and *Dof4* genes were selected randomly for self-pollination, and seeds were harvested. Ten T_1_ progeny of each mutant line were detected using Sanger sequencing. Mutations that occurred in the T_0_ generation were passed successfully to the next generation at a rate of 100% (Supplementary Table 2). Based on PCR amplification analysis of the T-DNA region of all T_1_ generations, the proportion of progeny without transgenes was in the range of 20–30% (Supplementary Table 2). To analysis the off-target effects caused by CRISPR/Cas9, four top-ranked off-target sites of all the sgRNAs used in *S*. *italica* were predicted by using CRISPR-P (Liu et al., 2017). All the putative off-target sites contained three to four mismatches against the on-target sites (Supplementary Table 3). All the T_0_ mutant plants that we obtained were analyzed using Sanger sequencing and no off-target effect was found.

### Efficient Multiplex Genome Editing System for Foxtail Millet

**FIGURE 2:**
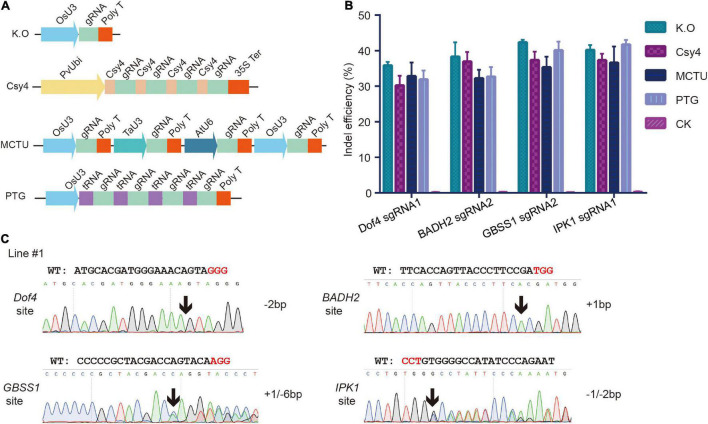
Multiples genome editing in foxtail millet using the CRISPR/Cas9 system. **(A)** Different strategies for simultaneously expressing multiple single guide RNA (sgRNAs). **(B)** Comparison of mutagenesis frequencies at four sgRNA target sites induced by single knockout constructs and multiple (MCTU-, tRNA-, and Csy4- based) knockout constructs in protoplast assay. **(C)** Sanger sequence chromatograms of quadruple mutations at the *Dof4*, *BADH2*, *GBSS1*, and *IPK1* target sites of representative T_0_ plants. The positions in which indels occurred were indicated by black arrows.

For multiplex genome editing in plants, sgRNAs can be transcribed from several independent promoters or from a single polycistronic cassette by a single promoter (Cermak et al., 2017). Endonuclease Csy4 and transfer RNA are two RNA cleavage elements in the polycistronic cassette (Tsai et al., 2014; Xie et al., 2015). To achieve multiplexed gene editing in *S*. *italica*, we tested the MCTU, Csy4, and PTG systems using a protoplast transfection assay. For MCTU method, individual sgRNA cassettes were driven by different Pol III promoters: OsU3, TaU3, and AtU6; for Csy4 method, single transcript with sgRNAs separated by Csy4 hairpins was driven by PvUbi promoter; for PTG method, single polycistronic tRNA-sgRNA transcript was driven by the OsU3 promoter (Figure 2A). The multiplex knockout constructs that targeted the *Dof4*, *BADH2*, *GBSS1*, and *IPK1* genes simultaneously were delivered into protoplasts. The PCR/T7EI method test results showed that all four target sites had mutations (Supplementary Figure 5). Next, NGS was performed to analyze the mutation efficiency and profiles (Supplementary Tables 1, 4). High indel mutagenesis efficiencies were observed at each target site, which ranged from 30.2 to 45.6%, this was comparable with that generated by single knockout constructs (Figure 2B). These results indicated that all the multiplex knockout systems worked effectively in *S*. *italica* protoplasts. Subsequently, MCTU system was chosen to generate multiple KO events. The MCTU vector was delivered into callus cells using an *Agrobacterium*-mediated transformation to create stable heritable mutants in the four target genes. In the T_0_ generation, 4 out of 5 recovered plants contained quadruple mutations and the remaining one contained triple mutations (Figure 2C).

### Achieved Effective Single-Base Editing in Foxtail Millet

**FIGURE 3:**
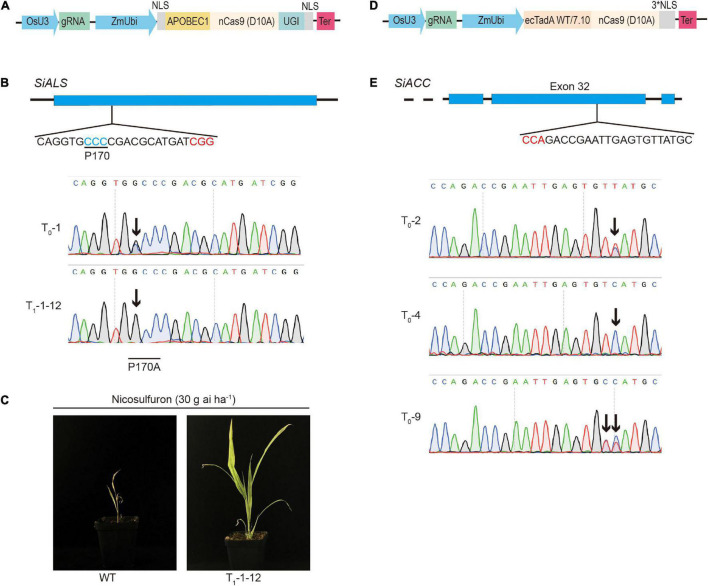
Targeted nucleotide substitutions using cytosine and adenine base editors. **(A)** Schematic review of the cytosine base editor used in this study. **(B)** Targeted mutagenesis in the *SiALS* gene induced by CBE. The PAM sequence and P170 amino acid that conferred herbicide resistance were highlighted in red and blue, respectively. Sanger sequencing was used to analyze the T_0_ transgenic plants. The mutated bases were indicated by black arrows. **(C)** Phenotypes of the homozygous P170A plants treated by nicosulfuron herbicide. **(D)** Schematic review of the adenine base editor used in this study. **(E)** Targeted mutagenesis in *SiACC* gene induced by ABE.

Several base editors have recently been developed to directly introduce precise nucleotide substitutions in endogenous genes. We explored further whether the base editors work efficiently in foxtail millet. BE3 was the most widely used cytosine base editors, which consisted of the rat APOBEC1 deaminase fused with a Cas9 nickase and an uracil glycosylase inhibitor (UGI), introduced C:G to T:A transition into targeted sites (Komor et al., 2016). We codon-optimized the BE3 cassette and cloned it into the pHUE411 backbone to generate the CBE vector (Figure 3A). Previous studies found that the missense mutation that occurred in the *OsALS* gene in the P171 amino acid, such as P171S, P171F, and P171A, conferred different levels of herbicide resistance (Kuang et al., 2020; Zhang et al., 2020). By sequence alignment, we selected the P170 amino acid of the *SiALS* (*Seita*.*1G169700*) gene, which was homologous to OsALS-P171, as the candidate target site to test the cytosine base editors in foxtail millet. sgRNA was designed to target the SiALS-P170 site, and the spacer sequence was inserted into the CBE vector, generating ALS-P170-CBE (Figure 3B). The resulted construct was firstly tested in the transient protoplast assay. We performed deep amplicon sequencing to analysis the mutations. We found C to T conversions at positions 7–9 within the protospacer, with efficiencies of 0.01–0.14%. We also observed C7 to G7 byproduct at the target site (Supplementary Figure 6A). Then the ALS-P170-CBE vector was delivered into the callus cells of foxtail millet (cv *xiaomi*) through an *Agrobacterium*-mediated transformation. In T_0_ generation, one line (T_0_-1) with a non-canonical C to G mutation was obtained, which resulted in a P170A missense mutation (Figure 3B and Table 1). No sgRNA-dependent off-target mutations were detected (Supplementary Table 3). The mutant line was then selfed and seeds from this line were harvested. The T_1_ homozygous, transgene-free P170A mutated plants were identified by PCR and Sanger sequencing. To test whether the P170A missense mutation conferred herbicide resistance, the homozygous T_1_ mutants were sprayed with the ALS inhibitor nicosulfuron. Four weeks later, the P170A mutants exhibited normal phenotypes, but the wild-type plants almost died (Figure 3C). These results indicated that missense mutations at the SiALS-P170 site conferred herbicide tolerance in foxtail millet.

Adenine base editors, which consisted of dimerized wild-type and evolved bacterial tRNA adenine deaminase TadA (ABE7.10) fused with a Cas9 nickase, introduced A:T to G:C transition into targeted sites (Gaudelli et al., 2017). We codon-optimized the ABE7.10 cassette and cloned it into the pHUE411 backbone to generate the ABE vector (Figure 3D). Previous studies showed that the C2186R mutation in the *OsACC* gene conferred tolerance to haloxyfop-R-methyl herbicide in rice (Li et al., 2018). To establish the adenine base editing system in foxtail millet, we chose the *SiACC* (*Seita*.*7G030200*) gene as the potential candidate target gene, and one sgRNA located at 32th exon was selected to target the C2088R amino acid, which corresponded to OsACC-C2186. The spacer sequence of the designed sgRNA was inserted into the ABE vector, generating ACC-C2186-ABE (Figure 3E). We observed A:T to G:C mutations at positions 3–8, with the efficiencies of 5.16–7.77%, in the transient protoplast assay (Supplementary Figure 6B). The ACC-C2186-ABE vector was then introduced into embryogenic callus cells by an *Agrobacterium*-mediated transformation as above, and 13 T_0_ independent transgenic lines were generated. Sanger sequencing revealed that four mutants that carried A●T to G●C conversion were recovered with a mutation frequency of 30.8%. Three lines of them carried homozygous or heterozygous edits at position T5, and the other one carried heterozygous edits at position T5 and T6 (Figure 3E and Table 1). No desired C2088R missense mutation were obtained. Further efforts are still ongoing to produce the desired T8 to C8 mutations. We did not detect mutations in potential off-target regions (Supplementary Table 3). These results at least indicated that the adenine base editor was effective in inducing targeted nucleotide substitution in foxtail millet.

## Discussion

In recent years, genome editing toolboxes have developed rapidly and they have been applied in various plant species. Here, a highly efficient genome editing system to targeted indels or nucleotide substitution in foxtail millet was established based on *Agrobacterium*-mediated editing reagent delivery. We successfully edited five endogenous genes using CRISPR/Cas9 and two endogenous genes using cytosine or adenine base editors in two varieties (Yugu1 and *xiaomi*). Mutations were heritable and could be transmitted faithfully to the next generation. In addition, we obtained one line with missense mutation P170A at the *ALS* gene that conferred herbicide resistance in foxtail millet. To our knowledge, this is the first study to demonstrate targeted mutagenesis in foxtail millet using base editing systems.

CRISPR/Cas9 induced DNA double-strand breaks at target loci is the most effective way to introduce specific genomic modifications. A transient protoplast assay enabled us to quickly test the mutation efficiency and pattern induced by genome editing reagents. We screened sgRNA, which had a higher targeted mutagenesis frequency for the same targeting gene in transient assay for the subsequent *Agrobacterium*-mediated transformation. In most cases, the mutation efficiencies in the T_0_ generation were 100%, which was much higher than that reported previously in foxtail millet (Cheng et al., 2021). In addition, all the mutants we obtained were homozygous or bi-allelic (Table 1), which indicated that the current system enabled us to generate heritable knockout mutant even if a small number of transgenic plants were recovered.

In this study, we also performed Illumina sequencing to analyze the mutation patterns induced by CRISPR/Cas9 at five target sites. For *BADH2* sgRNA2 and *IPK1* sgRNA1, the predominant mutation types were single nucleotide deletion or insertion, which was consisted with previous studies in other species. However, for *FMBP* sgRNA2, *Dof4* sgRNA1, and *GBSS1* sgRNA2, two or three nucleotide deletions occurred most frequently (Supplementary Figure 3). These results indicated that the preference mutation pattern seemed to be related not only to CRISPR/Cas9, but also to the genomic context and the target sequence. The amplicon deep sequencing analysis of mutations that occurred in protoplasts showed that 2.72–16.11% of the insertion mutations were >10 bp and derived from the plasmid (Figure 1C and Supplementary Figure 4). Genome-edited crops without any foreign DNA sequence were the ideal products to avoid strict regulation (Li et al., 2019). As we described previously in bread wheat, CRISPR/Cas9 delivered as *in vitro* transcripts (IVTs) or ribonucleoprotein complex (RNPs) was another effective method to reduce off-target effects and avoid foreign DNA insertions in genome-edited crops (Liang et al., 2017, 2018). Further efforts will be forthcoming to establish a DNA-free genome editing system in foxtail millet.

The base editing systems are new types of gene editing technology, which can realize the irreversible replacement of a single base at a specific site of a gene to promote the improvement of agricultural crops. In this study, we established both CBE and ABE systems successfully in foxtail millet, and we found that the base editing efficiencies were relatively lower when compared with the CRISPR/Cas9 knockout system (Table 1). We used CBE to target the P170 locus of the *SiALS* gene of foxtail millet. In theory, CBE can cause the substitution of base C to T. Under different circumstances, it can also realize the substitution of C to G or C to A. Previous studies reported that CBE produced multiple mutation types of OsALS-P171, which included P171S (C7 to T7), P171F (C7C8 to T7T8 or C7C8C9 to T7T8T9), P171A (C7 to G7), and P171Y (C7C8 to T7A8). All the four missense mutations that occurred at OsALS-P171 conferred herbicide tolerance in rice (Kuang et al., 2020). In our experiments, we only obtained one mutant line that carried P170A mutation, mainly because few T_0_ transgenic plants were obtained (Table 1). The genetic transformation system of foxtail millet still has much room for improvement. Plant regeneration is one of the limiting factors. It has been demonstrated that fine-tuning developmental genes, such as *BABY BOOM* (*BBM*), *WUSCHEL* (*WUS*), and GRF-GIF chimeras improved the regeneration efficiency of explant (Lowe et al., 2018; Debernardi et al., 2020). Hopefully these regeneration booster genes can also improve the transformation efficiency of foxtail millet when they are eventually implemented into the current genome editing system. Recently, several cytosine deaminases with various editing features have been utilized in CBE. BE3 with rAPOBEC1 prefer to edit TC not GC sequence motif with 3–9 editing window within the protospacer (Zong et al., 2017). While the PmCDA1 and hAID deaminases show no such sequence motif preference (Shimatani et al., 2017; Ren et al., 2018). Base editing based on human APOBEC3A exhibit high editing efficiency with broad editing window from 1 to 17 within the protospacer (Zong et al., 2018). Rationally designed human APOBEC3B showed remarkable precision and specificities in plants (Jin et al., 2020). In further experiments, protoplast assay could be used to evaluate the several deaminases at the same target and select the most effective one for stable transformation.

In the adenine base editing experiments, we chose to target the C2088R site of the *SiACC* gene of foxtail millet. Previous studies found that the C2168R (T to C) missense mutation in the *OsACC* gene conferred resistance to the herbicide haloxyfop-R-mithyl (Zhang et al., 2020). Although we successfully achieved the replacement of T5 and T6 positions, no base replacement occurred at the ideal T8 position, and no herbicide-resistant mutant was obtained (Figures 3B,E). This may be due to factor that base editors preferred to edit nucleotides at certain positions within the editing window. Recently, near-PAMless SpRY Cas9 variant was developed, which can be used to break through the limitations of PAM, adjust the appropriate editing window and preferences, and achieve precise editing of specific sites (Walton et al., 2020). In future experiments, more sgRNAs together with PAMless Cas9 variants can be designed and analyzed by the transient protoplast assay. The most effective one can be used for stable transformation to generate certain nucleotide substitution.

In summary, we have applied CRISPR/Cas9 gene editing technology and base editors successfully in foxtail millet. A protoplast-based system was developed to quickly evaluate the mutagenesis frequency and pattern of each target site in *S*. *italica* and a robust transformation system was implemented in local elite cultivars that produced stable mutated plants carrying targeted indels or nucleotide substitutions with high mutation frequency. We believe that valuable outcomes for foxtail millet genetic study and trait improvement for precise molecular breeding programs of foxtail millet can be achieved based on our system.

## Data Availability Statement

The datasets presented in this study can be found in online repositories. The names of the repository/repositories and accession number(s) can be found in the article/Supplementary Material.

## Author Contributions

ZL conceived and designed the project and wrote the manuscript with assistance from other authors. YW, ZL, and YR performed most of the experiments with assistance from LM and YG. YW, LM, YG, ZL, and YR analyzed experiments, read, and approved the manuscript.

## Conflict of Interest

YR was employed by Genovo Biotechnology Co. Ltd. The remaining authors declare that the research was conducted in the absence of any commercial or financial relationships that could be construed as a potential conflict of interest.

## Publisher’s Note

All claims expressed in this article are solely those of the authors and do not necessarily represent those of their affiliated organizations, or those of the publisher, the editors and the reviewers. Any product that may be evaluated in this article, or claim that may be made by its manufacturer, is not guaranteed or endorsed by the publisher.

## Footnotes

1
http://crispr.hzau.edu.cn/CRISPR2/

2
http://www.rgenome.net/cas-analyzer/#!
