# Supplementary material for: Efficient Genome Editing in Setaria italica Using CRISPR/Cas9 and Base Editors
Source: Front Plant Sci. 2022 Jan 13;12:815946. doi: 10.3389/fpls.2021.815946 (PMC8793480; doi:10.3389/fpls.2021.815946)
Supplement: Supplementary file 1 [file Data_Sheet_1.docx]

**Supplementary Material**

**Supplementary Figure 1.** Protoplast cells isolated from young stems and sheaths and transformed with the GFP reporter plasmids.

**Supplementary Figure 2.** Targeted mutagenesis of each target site in *S.* *italic* protoplasts analyzed by PCR/T7EI assay.

**Supplementary Figure 3.** Ten most frequent occurred mutation types induced by CRISPR/Cas9 at five sgRNA target sites in protoplasts.

**Supplementary Figure 4.** Insertion induced by CRISPR/Cas9 at two sgRNA target sites in protoplasts.

**Supplementary Figure 5.** PCR-T7EI analysis of mutagenesis frequencies at four sgRNA target sites induced by single knockout construct and multiple (MCTU-, tRNA- and Csy4- based) knockout construct in protoplast assay.

**Supplementary Figure 6.** Activities of base editors in foxtail millet protoplasts.

**Supplementary Table 1.** PCR primers used in this study and their applications.

**Supplementary Table 2**. Summary of T1 plant characterization, including mutation transmission and transgene-free ratio.

**Supplementary Table 3**. Potential off-target sites for sgRNAs used.

**Supplementary Table 4**. Illumina sequencing results of targeted mutations in foxtail millet protoplast.


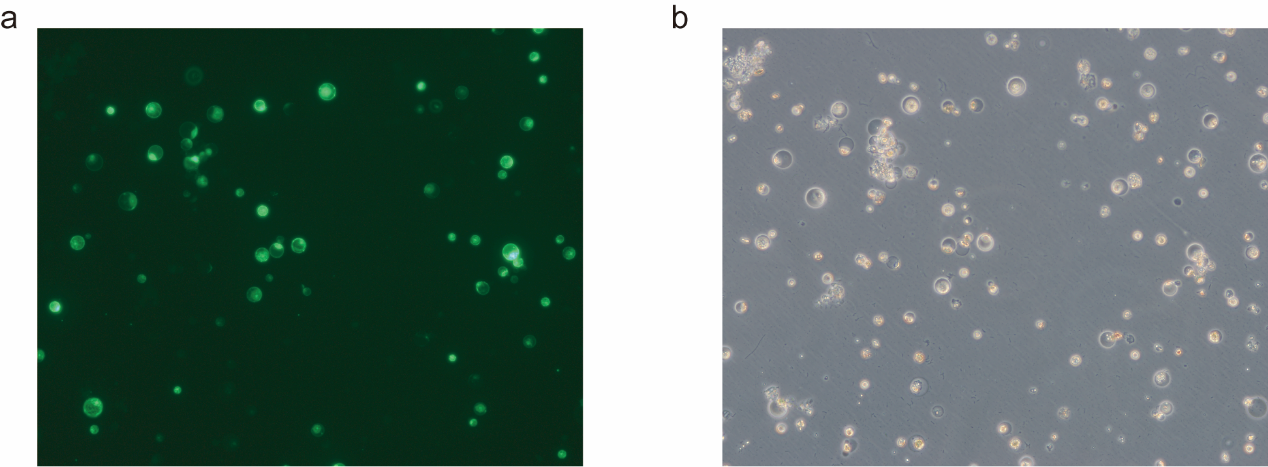


**Supplementary Figure 1.** Protoplast cells isolated from young stems and sheaths and transformed with the GFP reporter plasmids.


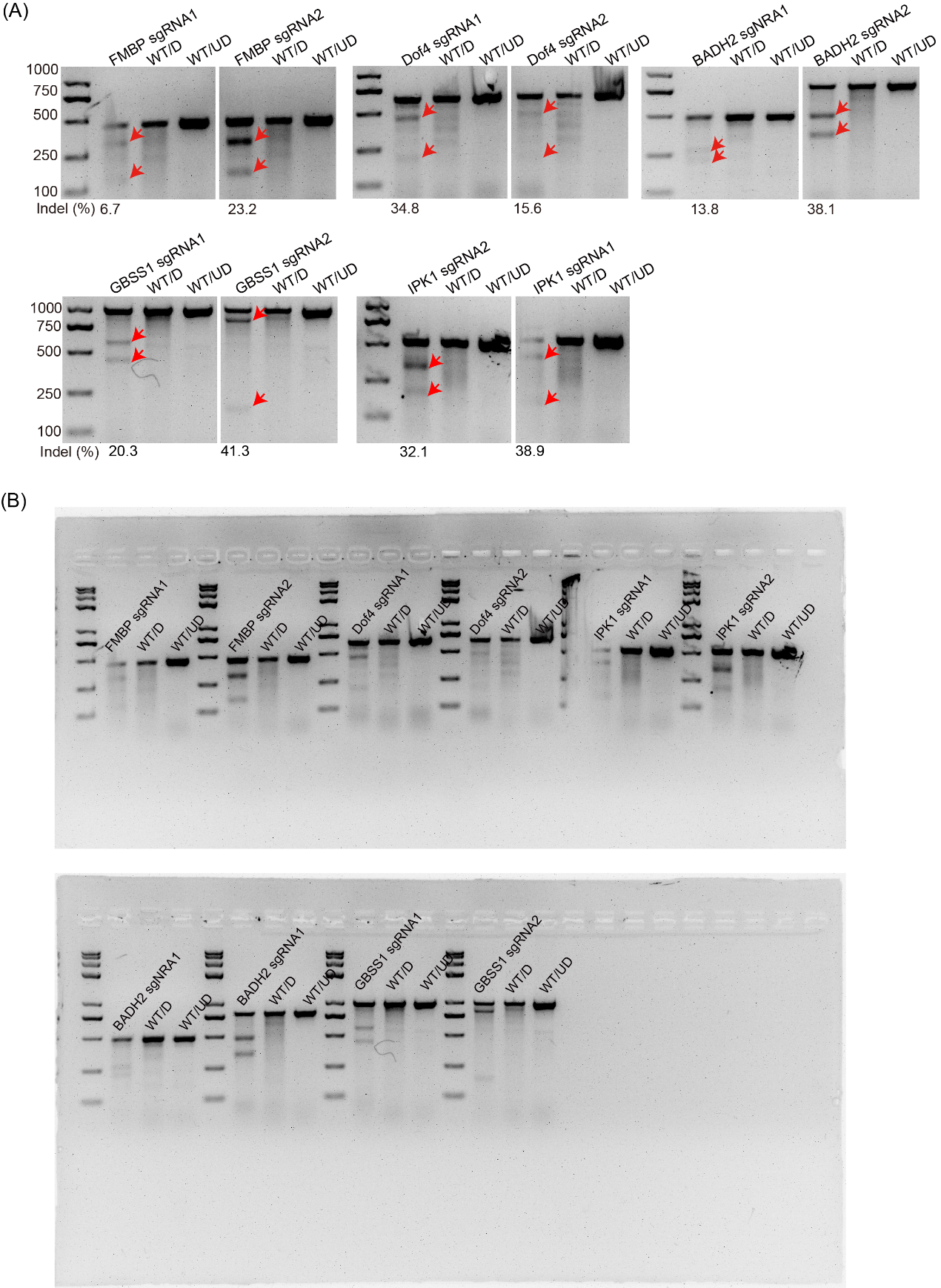


**Supplementary Figure 2.** Targeted mutagenesis of each target site in *S. italic* protoplasts analyzed by PCR/T7EI assay. (A) Mutation bands are indicated by red arrows. Mutagenesis frequencies are determined by Image J. WT/D and WT/U indicate wild type PCR amplicons with or without T7EI digestion. (B) Full scans of gel images presented in the panel A.


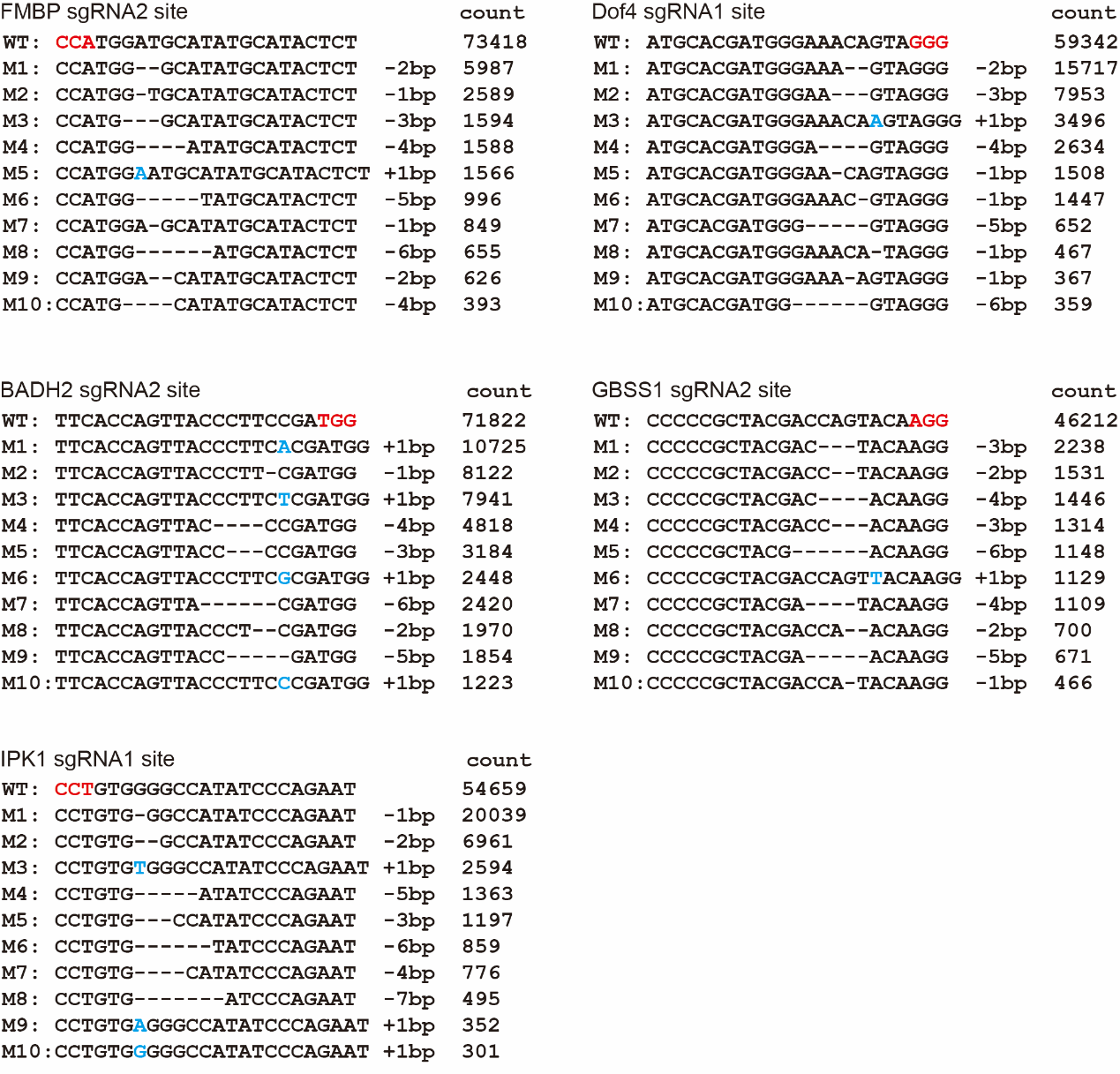


**Supplementary Figure 3.** Ten most occurred mutation types occurred at five sgRNA target sites in protoplasts. Mutations are detected by deep amplicon sequencing. The count indicates the occurrence frequency of each mutation type. The PAM sequence and insertion nucleotide were highlighted in red and blue, respectively.


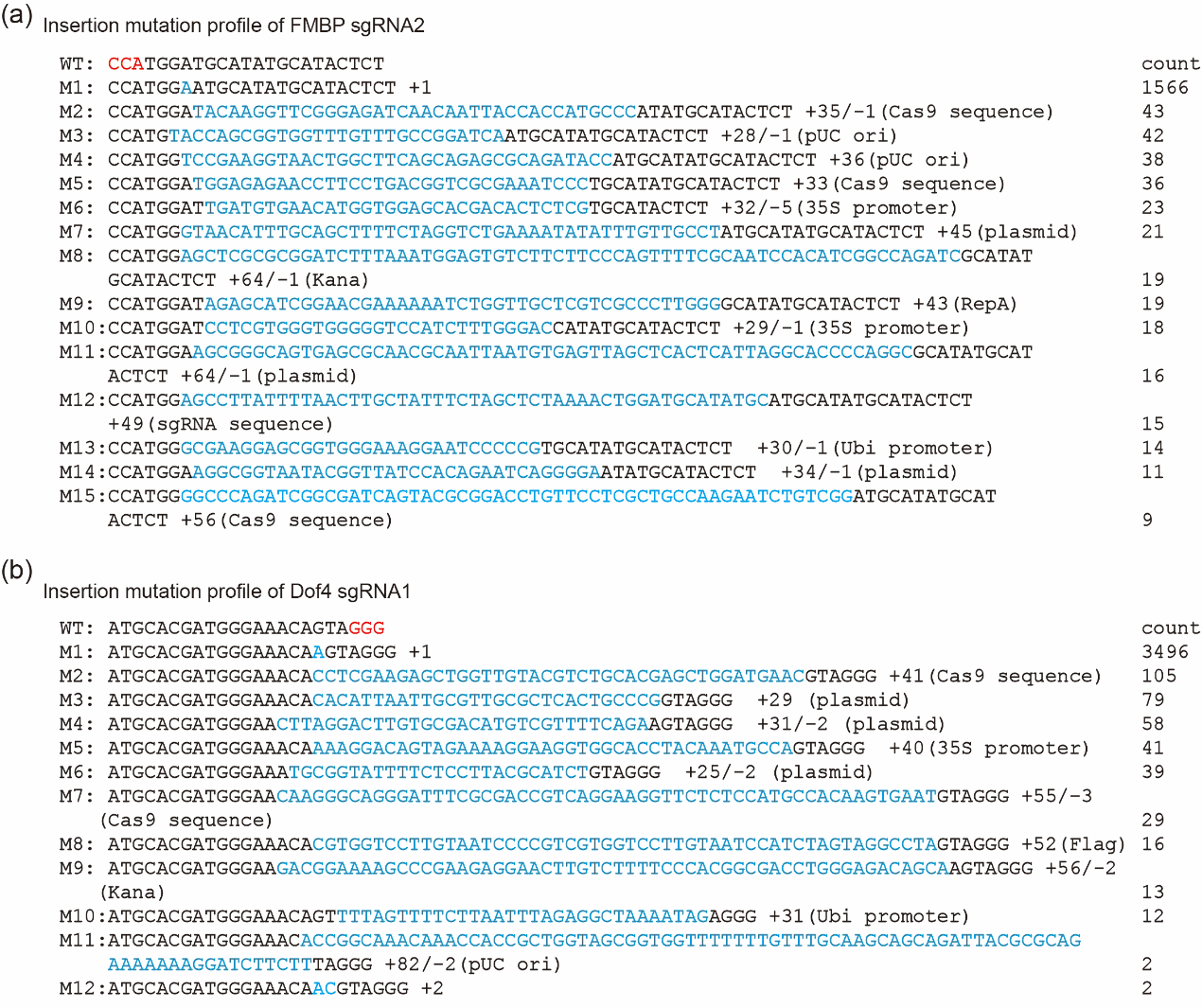


**Supplementary Figure 4.** Insertion induced by CRISPR/Cas9 at two target sites in protoplasts. Insertion mutation profile of FMBP sgNRA2 and Dof4 sgRNA1 target sites are shown. Mutations are detected by deep amplicon sequencing. The count indicates the occurrence frequency of each mutation type. Insertion sequences with more than ten base pairs are aligned with the CRISPR/Cas9 plasmids. The PAM sequence and insertion nucleotide were highlighted in red and blue, respectively.


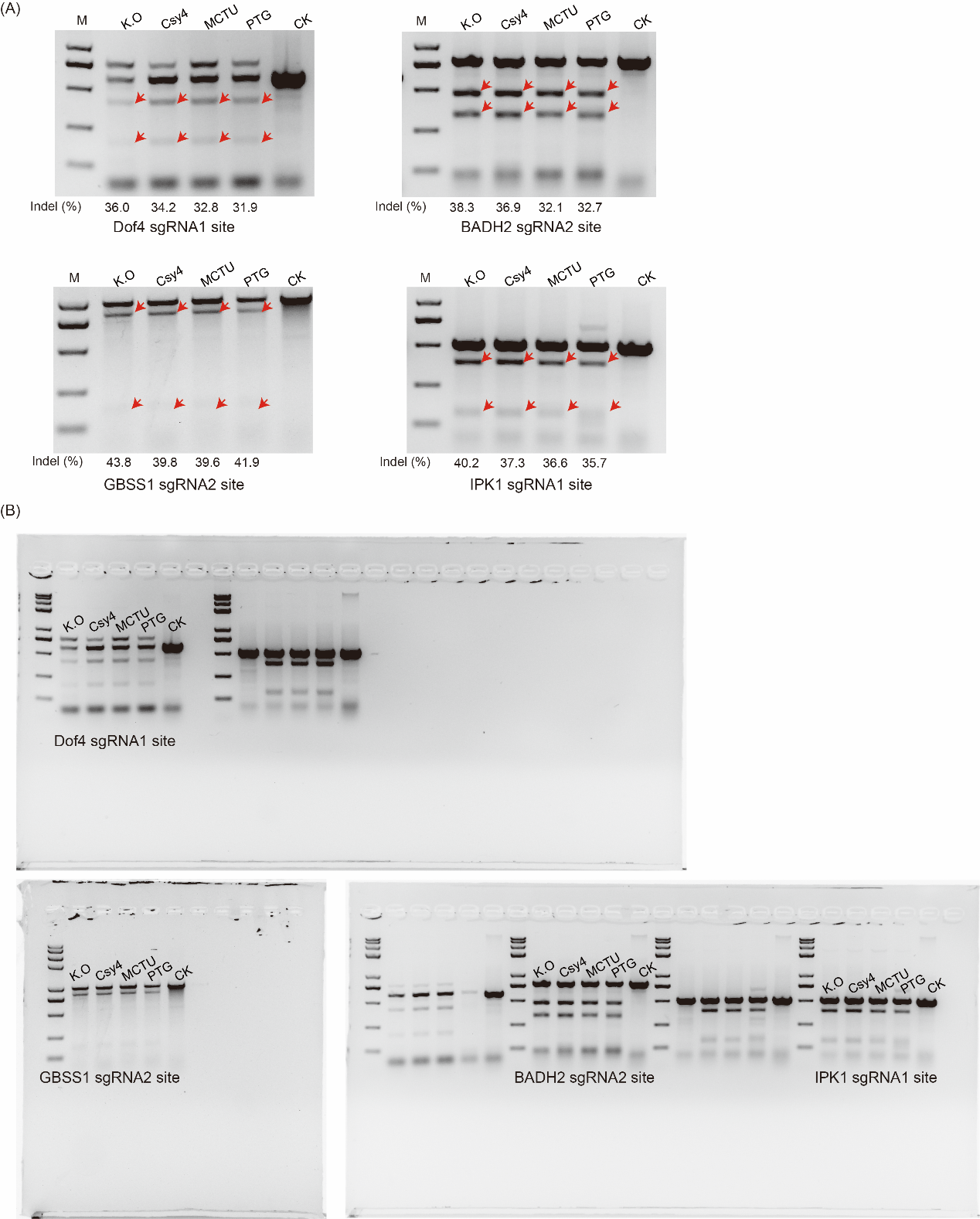


**Supplementary Figure 5.** PCR-T7EI analysis of mutagenesis frequencies at four sgRNA target sites induced by single knockout construct and multiple (MCTU-, tRNA- and Csy4- based) knockout construct in protoplast assay. (A) Mutation bands are indicated by red arrows. Mutagenesis frequencies are determined by Image J. (B) Full scans of gel images presented in the panel A.

**
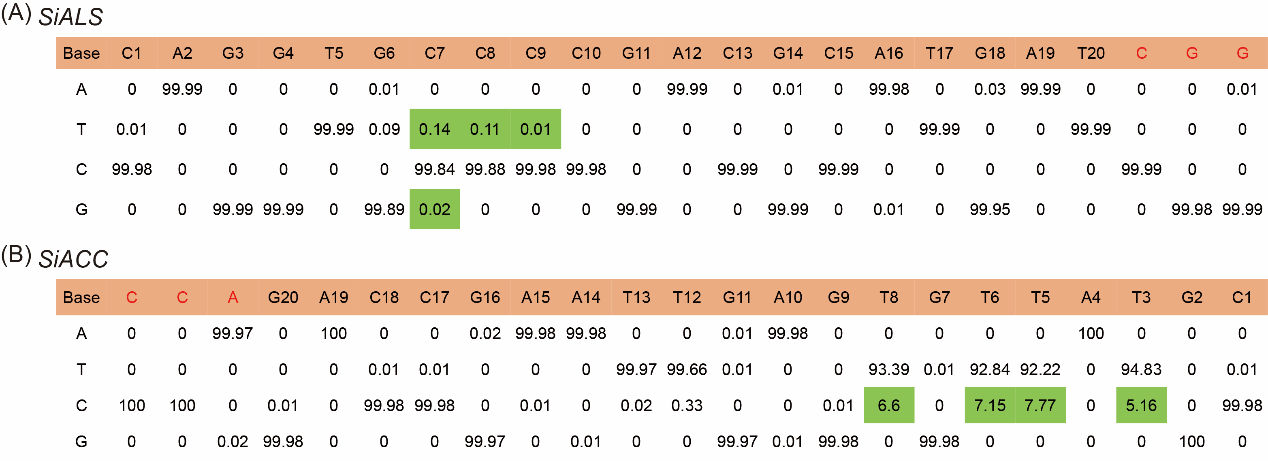
**

**Supplementary Figure 6.** Activities of base editors in foxtail millet protoplasts. (A) CBE mediated C:G to T:A conversions in *SiALS* gene. (B) ABE mediated A:T to G:C conversions in *SiACC* gene. The PAM sequence and targeted mutations were highlighted in red and green, respectively.

**Supplementary Table 1**. PCR primers used in this study and their applications.

| Primer name | Primer sequence | Application |
| --- | --- | --- |
| MT1-Si9-BsF  MT1-Si9-F0  MT0-BsR2 | ATATATGGTCTCTGGCGTGCACGATGGGAAACAGTAGTT  TGTGCACGATGGGAAACAGTAGTTTTAGAGCTAGAAATAGC  ATTATTGGTCTCTGCTTCTTGGTGCCGCgcct | Primers used for MCTU plasmid construction |
| MT2-Si12-BsF2  MT2-Si12-F0  MT0-BsR3 | ATATATGGTCTCTAAGCGTCACCAGTTACCCTTCCGAGTT  TGTCACCAGTTACCCTTCCGAGTTTTAGAGCTAGAAATAGC  ATTATTGGTCTCTCAATCACTACTTCGACTCTAGC | Primers used for MCTU plasmid construction |
| MT3-Si14-BsF3  MT3-Si14-F0  MT4-Si15-R0  MT4-Si15-BsR | ATATATGGTCTCTATTGCCCCGCTACGACCAGTACAGTT  TGCCCCGCTACGACCAGTACAGTTTTAGAGCTAGAAATAGC  AACGTGGGGCCATATCCCAGAACGCCACGGATCATCTGCACAACT  ATTATTGGTCTCTAAACGTGGGGCCATATCCCAGAAC | Primers used for MCTU plasmid construction |
| L5AD5-F  gRSi9-R | GCAGGATGGGCAGTCTGGGCAACAAAGCACCAGTGG  ATGGTCTCACCCATCGTGCATtgcaccagccgggaa | Primers used for PTG plasmid construction |
| gRSi9-F  gRSi12-R | TAGGTCTCCTGGGAAACAGTAgttttagagctagaa  ATGGTCTCAGTAACTGGTGAAtgcaccagccgggaa | Primers used for PTG plasmid construction |
| gRSi12-F  gRSi14-R | TAGGTCTCCTTACCCTTCCGAgttttagagctagaa  ATGGTCTCATCGTAGCGGGGGtgcaccagccgggaa | Primers used for PTG plasmid construction |
| gRSi14-F  gRSi15-R | TAGGTCTCCACGACCAGTACAgttttagagctagaa  ATGGTCTCAATATCCCAGAATtgcaccagccgggaa | Primers used for PTG plasmid construction |
| gRSi15-F  L3AD5-R | TAGGTCTCCATATGGCCCCACgttttagagctagaa  AACGGATGAGCGACAGCAAACAAAAAAAAAAGCACCGACTCG | Primers used for PTG plasmid construction |
| S5AD5-F  S5AD5-R | GCAGGATGGGCAGTCTGGGCA  AACGGATGAGCGACAGCAAAC | Primers used for PTG plasmid construction |
| oPvUbi1  CSY_gRNASi9 | TGCTCTTCGCGCCACGTCAGTGTTTGGTTTCC  TATCACCTGCCCCCCCCATCGTGCATCTGCCTATACGGCAGTGAAC | Primers used for Csy4 plasmid construction |
| REP_gRNASi9  CSY_gRNASi12 | TATCACCTGCCCCATGGGAAACAGTAGTTTTAGAGCTAGAAATAGC  TATCACCTGCCCCCGTAACTGGTGAACTGCCTATACGGCAGTGAAC | Primers used for Csy4 plasmid construction |
| REP_gRNASi12  CSY_gRNASi14 | TATCACCTGCCCCATTACCCTTCCGAGTTTTAGAGCTAGAAATAGC  TATCACCTGCCCCCTCGTAGCGGGGGCTGCCTATACGGCAGTGAAC | Primers used for Csy4 plasmid construction |
| REP_gRNASi14  CSY_gRNASi15 | TATCACCTGCCCCAACGACCAGTACAGTTTTAGAGCTAGAAATAGC  TATCACCTGCCCCCATATCCCAGAATCTGCCTATACGGCAGTGAAC | Primers used for Csy4 plasmid construction |
| REP_gRNASi15  CSY_term | TATCACCTGCCCCAATATGGCCCCACGTTTTAGAGCTAGAAATAGC  TGCTCTTCTGACCTGCCTATACGGCAGTGAAC | Primers used for Csy4 plasmid construction |
| D-FMBP-F  D-FMBP-R | TTCACTGACTCACCAGAACGC CGCTGCCACTACTCCTACTCG | Amplifying the FMBP sgRNA1 and sgRNA2 target site and 1^st^ PCR for deep sequencing |
| D-Dof4-F  D-Dof4-R | GTTCAGAAACGGAAGGCGAAG AACTTGGTGTTGGTGGACTCG | Amplifying the Dof4 sgRNA1 and sgRNA2 target site and 1^st^ PCR for deep sequencing |
| D-BADH2-1F  D-BADH2-1R | ATCCCACCAACCCAAACCACT TGACACGCTCAATCCATTTCG | Amplifying the BADH2 sgRNA1 target site |
| D-BADH2-2F  D-BADH2-2R | CCTTGTAATGTTTGGGTCATC GGACAGCAGTAGAATAGTGGG | Amplifying the BADH2 sgRNA2 target site and 1^st^ PCR for deep sequencing |
| D-GBSS1-F  D-GBSS1-R | ACTCACAAGTCAGAAGCACCC GGACGATGAATGAAATCTCAC | Amplifying the GBSS1 sgRNA1 and sgRNA2 target site and 1^st^ PCR for deep sequencing |
| D-IPK-F  D-IPK-R | GTGTCCTCATTTGCTTGGTGT GAATAAGTTGAATCCGAACCG | Amplifying the IPK sgRNA1 and sgRNA2 target site and 1^st^ PCR for deep sequencing |
| BA1-Si8-F  BA2-Si8-R | CGATGTACTCCATCGACAAAGCTAGC  TGACCACGCTGCAGTGATCTGATCAC | 2^nd^ PCR for deep sequencing of FMBP sgRNA2 site induced by single knockout |
| BA1-Si15F  BA2-Si15R | CGATGTCTTGGCAAGGTACTGCGGGT  TGACCAACCATCAACATGATTAGCAC | 2^nd^ PCR for deep sequencing of IPK1 sgRNA1 site induced by single knockout |
| BA3-Si15F  BA4-Si15R | ACAGTGCTTGGCAAGGTACTGCGGGT  GCCAATACCATCAACATGATTAGCAC | 2^nd^ PCR for deep sequencing of IPK1 sgRNA1 site induced by Csy4 based multiple knockout |
| BA5-Si15F  BA6-Si15R | CAGATCCTTGGCAAGGTACTGCGGGT  CTTGTAACCATCAACATGATTAGCAC | 2^nd^ PCR for deep sequencing of IPK1 sgRNA1 site induced by MCTU based multiple knockout |
| BA7-Si15F  BA8-Si15R | ATCACGCTTGGCAAGGTACTGCGGGT  TTAGGCACCATCAACATGATTAGCAC | 2^nd^ PCR for deep sequencing of IPK1 sgRNA1 site induced by PTG based multiple knockout |
| BA9-Si15F  BA10-Si15R | ACTTGACTTGGCAAGGTACTGCGGGT  GATCAGACCATCAACATGATTAGCAC | 2^nd^ PCR for deep sequencing of IPK1 sgRNA1 site of wild type |
| BA11-Si14F  BA12-Si14R | TAGCTTCATCGTGTCATTGGTGCATG  GGCTACTTGTCAGCGAGAGATGGACA | 2^nd^ PCR for deep sequencing of GBSS1 sgRNA2 site induced by single knockout |
| BA13-Si14F  BA14-Si14R | AGTCAACATCGTGTCATTGGTGCATG  AGTTCCTTGTCAGCGAGAGATGGACA | 2^nd^ PCR for deep sequencing of GBSS1 sgRNA2 site induced by Csy4 based multiple knockout |
| BA15-Si14F  BA16-Si14R | ATGTCACATCGTGTCATTGGTGCATG  CCGTCCTTGTCAGCGAGAGATGGACA | 2^nd^ PCR for deep sequencing of GBSS1 sgRNA2 site induced by MCTU based multiple knockout |
| BA17-Si14F  BA18-Si14R | GTAGAGCATCGTGTCATTGGTGCATG  GTCCGCTTGTCAGCGAGAGATGGACA | 2^nd^ PCR for deep sequencing of GBSS1 sgRNA2 site induced by PTG based multiple knockout |
| BA19-Si14F  BA20-Si14R | GTGAAACATCGTGTCATTGGTGCATG  GTGGCCTTGTCAGCGAGAGATGGACA | 2^nd^ PCR for deep sequencing of GBSS1 sgRNA2 site of wild type |
| BA21-Si14F  BA22-Si14R | GTTTCGTGCCACTTCTTTTATCAGGA  CGTACGAAACAGTGAGATGGGGCAGA | 2^nd^ PCR for deep sequencing of BADH2 sgRNA2 site induced by single knockout |
| BA23-Si14F  BA24-Si14R | GAGTGGTGCCACTTCTTTTATCAGGA  GGTAGCAAACAGTGAGATGGGGCAGA | 2^nd^ PCR for deep sequencing of BADH2 sgRNA2 site induced by Csy4 based multiple knockout |
| BA25-Si14F  BA26-Si14R | ACTGATTGCCACTTCTTTTATCAGGA  ATGAGCAAACAGTGAGATGGGGCAGA | 2^nd^ PCR for deep sequencing of BADH2 sgRNA2 site induced by MCTU based multiple knockout |
| BA27-Si14F  BA28-Si14R | ATTCCTTGCCACTTCTTTTATCAGGA  CAAAAGAAACAGTGAGATGGGGCAGA | 2^nd^ PCR for deep sequencing of BADH2 sgRNA2 site induced by PTG based multiple knockout |
| BA29-Si14F  BA30-Si14R | CAACTATGCCACTTCTTTTATCAGGA  CACCGGAAACAGTGAGATGGGGCAGA | 2^nd^ PCR for deep sequencing of BADH2 sgRNA2 site of wild type |
| BA31-Si14F  BA32-Si14R | CACGATTCGTCGCTGATCAATGCATG  CACTCATAGCGGGTAAATATCTACAC | 2^nd^ PCR for deep sequencing of Dof4 sgRNA1 site induced by single knockout |
| BA33-Si14F  BA34-Si14R | CAGGCGTCGTCGCTGATCAATGCATG  CATGGCTAGCGGGTAAATATCTACAC | 2^nd^ PCR for deep sequencing of Dof4 sgRNA1 site induced by Csy4 based multiple knockout |
| BA35-Si14F  BA36-Si14R | CATTTTTCGTCGCTGATCAATGCATG  CCAACATAGCGGGTAAATATCTACAC | 2^nd^ PCR for deep sequencing of Dof4 sgRNA1 site induced by MCTU based multiple knockout |
| BA37-Si14F  BA38-Si14R | CGGAATTCGTCGCTGATCAATGCATG  CTAGCTTAGCGGGTAAATATCTACAC | 2^nd^ PCR for deep sequencing of Dof4 sgRNA1 site induced by PTG based multiple knockout |
| BA39-Si14F  BA40-Si14R | CTATACTCGTCGCTGATCAATGCATG  CTCAGATAGCGGGTAAATATCTACAC | 2^nd^ PCR for deep sequencing of Dof4 sgRNA1 site of wild type |

**Supplementary Table 2**. Summary of T1 plant characterization, including mutation transmission and transgene-free ratio.

| T_0_ plant ID | No. of T_1_ plants tested | No. of mutants, transmission ratio | T-DNA free, ratio |
| --- | --- | --- | --- |
| *fmbp* T_0_-1 | 10 | 10, 100% | 2, 20% |
| *fmbp* T_0_-2 | 10 | 10, 100% | 3, 30% |
| *fmbp* T_0_-3 | 10 | 10, 100% | 2, 20% |
| *dof4* T_0_-1 | 10 | 10, 100% | 2, 20% |
| *dof4* T_0_-2 | 10 | 10, 100% | 2, 20% |
| *dof4* T_0_-6 | 10 | 10, 100% | 3, 30% |

**Supplementary Table 3.** Potential off-target sites for sgRNAs used.

| Target site | Off-target site | sequence | Forward primer sequence | Reverse primer sequence |
| --- | --- | --- | --- | --- |
| *FMBP* sgRNA2 | WT | CCATGGATGCATATGCATACTCT |  |  |
|  | OT-1 | AGctcATGCATATGCATCCATGG | GCTCGTTGACTTGCCATAAG | CGTAGTTGCCGTAGTAGAAGG |
|  | OT-2 | AGtGcATGCATATGgATCCAGAG | AAATCGGTGAGAGAAACAGC | AGAGACCAGAACACAGAGTCC |
|  | OT-3 | AGAaTATGtATAaGCATCCACAG | CGGCTACAGTTGAGTTGAAG | CATTCGGAGATGACTAAAGGAT |
|  | OT-4 | AaAGaAaGCATcTGCATCCAAGG | TCTTGGGCTATGGCTTCACG | GTGCGTAATCTTCGGGTCCA |
| *Dof4* sgRNA1 | WT | ATGCACGATGGGAAACAGTAGGG |  |  |
|  | OT-1 | gTtCACtATGGGAAACAGTAAAG | GTTGTCGCCGAGTGTTTATC | AGCAAGTCCTTACCCTACCA |
|  | OT-2 | gTGCACtAcGGGAAACAGTAAAG | GTATTTGCCGAGTGCCAAGG | GTGCCGTTCTGGTGTGGAAT |
|  | OT-3 | cTGCACtAcGGGAAACAGTAAAG | CCAAGCATCTCGGCTACAAG | ATACTCGGCAAACCACCTCG |
|  | OT-4 | AgGCACtAccGGAAACAGTAAGG | ACACCCGAGGAGTAGAAGGC | TAGGAAAGTTCGCCAAGTGC |
| *BADH2* sgRNA2 | WT | TTCACCAGTTACCCTTCCGATGG |  |  |
|  | OT-1 | aTCACCAtTTAaCCTTCCcAAGG | CAAAGATGCTGGGTGAACAAA | GTGCAACCAAGGCTCCAAATA |
|  | OT-2 | cTCgCCAGTTcCCCaTCCGACGG | CTCTTTATGAGGTTGTGCGATTTT | CAGGCTGTAGGCTGCGTTGA |
|  | OT-3 | TTCtCCAGTTcCtCTTCCGtTGG | ATACAGGTAATCGGATGATGAGG | GAAAGGCTGAGGATGGAGCT |
|  | OT-4 | TTCACCgGTTctCCaTCCGACGG | GCCATTGTGGGTGAGGTTG | CTTGTCAATCGCACGCTCA |
| *GBSS1* sgRNA2 | WT | CCCCCGCTACGACCAGTACAAGG |  |  |
|  | OT-1 | CCCgCGCccCGACCAGaACAAAG | CGTTGGTGTCGGCGTGGTA | CAGCGAGCAGAGGCAGGAT |
|  | OT-2 | CCaCgGCcACGACCAGTtCAGGG | ATAGAACCCAGGCATCACC | ACGACAAACACCCGAAACT |
|  | OT-3 | CCCCCGCcACGgCaAGTACATGG | AACATAACAAACAGCCCAAGA | GAGGCCCAACTACTCCAAT |
|  | OT-4 | CaCCCGCaAgGACaAGTACAAAG | CAACCAACTAACGCCCACTG | TACAAACAACCGCAAGACGT |
| *IPK1* sgRNA1 | WT | CCTGTGGGGCCATATCCCAGAAT |  |  |
|  | OT-1 | ATgCTGGtATAgGGCCCaACAGG | GCTGCGTGATGAAGCGTGAA | CGGAGGGATAGGTCCTGTGAG |
|  | OT-2 | gaTCTGGGATATGGaCCCAaTGG | AGGTATAGTGCAGGATTTCCA | CAGTGATGACCGTCAAGACA |
|  | OT-3 | ATcCTaGGATATGGCCaCAtGGG | CTGGCTACTGTAAGCATCGTCG | GCTTCAAAGAGCGGAGACAC |
|  | OT-4 | AcTCTGGGcTcTGGCCCCgCCGG | TCAGATACGGACCGACAGGC | CGCTCAATGGGCTCAGAAGA |
| *ALS* sgRNA | WT | CAGGTGCCCCGACGCATGATCGG |  |  |
|  | OT-1 | CAtcTGaCCCGACGCATGATTGG | TAACTCCTTATCCGAAACCTC | TTACGAAACAACTTCAACACG |
|  | OT-2 | CAcGgGCCCCGACGCgTGATGAG | CTTGGCTGCTCACCATTTC | CTGCTCAGAGGCTCCGATA |
|  | OT-3 | gAGtTGCCaCGACaCATGATAGG | ACTAGAGGAAACGTCCAACAAA | AAGTGGCAGAAGAAGAAGAGC |
|  | OT-4 | CAGGTGCaCCaAgaCATGATCAG | CACAGACCATGCGTGCTGAA | TGCGGAGAATAGATTGATGAGAC |
| *ACC* sgRNA | WT | CCAGACCGAATTGAGTGTTATGC |  |  |
|  | OT-1 | cCgTgtCACTCAATTCGGTCAGG | ACTTCAGCGACGAGAACCCTA | ATGTCAGACACCGGCCTACT |
|  | OT-2 | aCATAACACTgAATTCaGTCCAG | AACTAGAACAGTGGAGCGAATA | AGATAAGAGGGCTACTTTGAGAT |
|  | OT-3 | GCtTAACcCTCgATTCGGTtTGG | GATCGGACAAATCCTTGATG | GTTCTCGTGGACTCGCACTC |
|  | OT-4 | cCATgtCACTCAATcCGGTCAGG | TTTCCCTTGTCCATTTCCTG | TACATAACGGCTCGGCTTGG |

Note: The PAM sequences are highlighted in red and the 5’-NAG-3’ PAM are also selected for off-target analysis. The mismatched nucleotides are indicated in lower case.

**Supplementary Table 4**. Illumina sequencing results of targeted mutations in foxtail millet protoplast.

| Target site | Vector | Repeat | Total reads | Deletion reads | Insertion reads | Indel ratio |
| --- | --- | --- | --- | --- | --- | --- |
| *Dof4* sgRNA1 | K.O | 1 | 101424 | 33351 | 4169 | 37.0% |
|  |  | 2 | 158609 | 52102 | 4064 | 35.4% |
|  |  | 3 | 104652 | 33872 | 2934 | 35.2% |
|  | Csy4 | 1 | 106983 | 28254 | 896 | 27.2% |
|  |  | 2 | 145657 | 44395 | 945 | 31.1% |
|  |  | 3 | 97376 | 30688 | 815 | 32.4% |
|  | MCTU | 1 | 77497 | 24320 | 1680 | 33.5% |
|  |  | 2 | 164510 | 43396 | 3731 | 28.6% |
|  |  | 3 | 91845 | 30729 | 2585 | 36.3% |
|  | PTG | 1 | 155809 | 50378 | 2565 | 34.0% |
|  |  | 2 | 202629 | 62071 | 4016 | 32.6% |
|  |  | 3 | 138659 | 38198 | 2152 | 29.1% |
| *BADH2* sgRNA2 | K.O | 1 | 131662 | 28231 | 25192 | 40.6% |
|  |  | 2 | 241622 | 33761 | 47335 | 33.6% |
|  |  | 3 | 123144 | 22965 | 27123 | 40.7% |
|  | Csy4 | 1 | 70178 | 18491 | 8957 | 39.1% |
|  |  | 2 | 200273 | 42304 | 25510 | 33.9% |
|  |  | 3 | 780377 | 178279 | 116721 | 37.8% |
|  | MCTU | 1 | 114997 | 21423 | 18588 | 34.8% |
|  |  | 2 | 187308 | 23749 | 32495 | 30.0% |
|  |  | 3 | 188109 | 25971 | 34091 | 31.9% |
|  | PTG | 1 | 128749 | 28099 | 12419 | 31.5% |
|  |  | 2 | 229663 | 52555 | 29656 | 35.8% |
|  |  | 3 | 119850 | 20659 | 16134 | 30.7% |
| *GBSS1* sgRNA2 | K.O | 1 | 118002 | 42614 | 6575 | 41.7% |
|  |  | 2 | 111925 | 40294 | 7012 | 42.3% |
|  |  | 3 | 142119 | 52192 | 9127 | 43.1% |
|  | Csy4 | 1 | 101659 | 37122 | 3319 | 39.8% |
|  |  | 2 | 123538 | 39118 | 4258 | 35.1% |
|  |  | 3 | 140540 | 47273 | 5064 | 37.2% |
|  | MCTU | 1 | 122791 | 38879 | 8177 | 38.3% |
|  |  | 2 | 145325 | 41932 | 9431 | 35.3% |
|  |  | 3 | 143294 | 37153 | 9107 | 32.3% |
|  | PTG | 1 | 129847 | 41316 | 7182 | 37.4% |
|  |  | 2 | 146724 | 51912 | 7856 | 40.7% |
|  |  | 3 | 177794 | 63045 | 11974 | 42.2% |
| *IPK1* sgRNA1 | K.O | 1 | 98772 | 35353 | 4063 | 39.9% |
|  |  | 2 | 168194 | 63982 | 6195 | 41.7% |
|  |  | 3 | 122198 | 43711 | 3824 | 38.9% |
|  | Csy4 | 1 | 130524 | 47458 | 3944 | 39.4% |
|  |  | 2 | 192238 | 65563 | 4883 | 36.6% |
|  |  | 3 | 41058 | 13584 | 1142 | 35.9% |
|  | MCTU | 1 | 75990 | 27507 | 3001 | 40.1% |
|  |  | 2 | 244135 | 83564 | 9942 | 38.3% |
|  |  | 3 | 121378 | 34297 | 3815 | 31.4% |
|  | PTG | 1 | 126305 | 47275 | 4394 | 40.9% |
|  |  | 2 | 113069 | 43879 | 4978 | 43.2% |
|  |  | 3 | 84401 | 31505 | 3150 | 41.1% |
